# Prurigo: review of its pathogenesis, diagnosis, and treatment^☆^

**DOI:** 10.1016/j.abd.2023.11.003

**Published:** 2024-03-16

**Authors:** Paulo Ricardo Criado, Mayra Ianhez, Roberta Fachini Jardim Criado, Juliana Nakano, Daniel Lorenzini, Hélio Amante Miot

**Affiliations:** a Centro Universitário ABC Faculty of Medicine, Santo André, SP, Brazil; b Faculdade de Ciências Médicas de Santos (Fundação Lusíada), Santos, SP, Brazil; c Department of Dermatology, Hospital de Doenças Tropicais de Goiás, Goiânia, GO, Brazil; d Alergoskin Alergia e Dermatologia, UCARE Center and ADCARE, Santo André, SP, Brazil; e Dermatology Outpatient Clinic, Santa Casa de Misericórdia de São Paulo, Brazil; f Irmandade Santa Casa de Misericórdia de Porto Alegre, Porto Alegre, RS, Brazil; g Department of Infectious Diseases, Dermatology, Imaging Diagnosis and Radiotherapy, Faculty of Medicine, Universidade Estadual Paulista, Botucatu, SP, Brazil

**Keywords:** Dermatitis, atopic, Drug therapy, Immunocompromised host, Janus kinase inhibitors, Prurigo, Pruritus

## Abstract

Prurigo is a reactive, hyperplastic skin condition characterized by pruritic papules, plaques, and/or nodules. The temporal classification includes acute/subacute and chronic disease (≥ 6 weeks), with different clinical variants, synonymies, and underlying etiological factors. The immunology of chronic prurigo shows similarities with atopic dermatitis due to the involvement of IL-4 and IL-13, IL-22, and IL-31. Treatment includes antihistamines, topical steroids, dupilumab, and JAK inhibitors. Several conditions manifest clinically as prurigo-like lesions, and the correct clinical diagnosis must precede correct treatment. Furthermore, chronic prurigos represent a recalcitrant and distressing dermatosis, and at least 50% of these patients have atopic diathesis, the treatment of which may induce adverse effects, especially in the elderly. The quality of life is significantly compromised, and topical treatments are often unable to control symptoms and skin lesions. Systemic immunosuppressants, immunobiologicals, and JAK inhibitors, despite the cost and potential adverse effects, may be necessary to achieve clinical improvement and quality of life. This manuscript reviews the main types of prurigo, associated diseases, their immunological bases, diagnosis, and treatment.

## Introduction

Prurigo is a reactive hyperplastic skin condition characterized by papules, plaques, and/or nodules, either isolated or multiple, with intense pruritus. Some authors classify prurigo according to the type (acute, subacute or chronic), or clinical form or causative agent/associated disease. There are many synonyms and descriptions of overlapping conditions under the same name, which makes it difficult to review the available information.^1^, ^2^, ^3^

The term “prurigo” originates from the Latin word *pruire*, which means itching, named by Ferdinand von Hebra, when characterizing papules and nodules with intense pruritus, in Vienna in 1850.^3^, ^4^ Other authors, however, attribute to Robert Willan more than 200 years ago, the description of pruritic papules under the term prurigo.^4^, ^5^

This manuscript reviews the main types of prurigo, associated diseases, their immunological bases, diagnosis, and treatment.

## Clinical forms of Prurigo

### Acute/subacute prurigo

**Figure 1:**
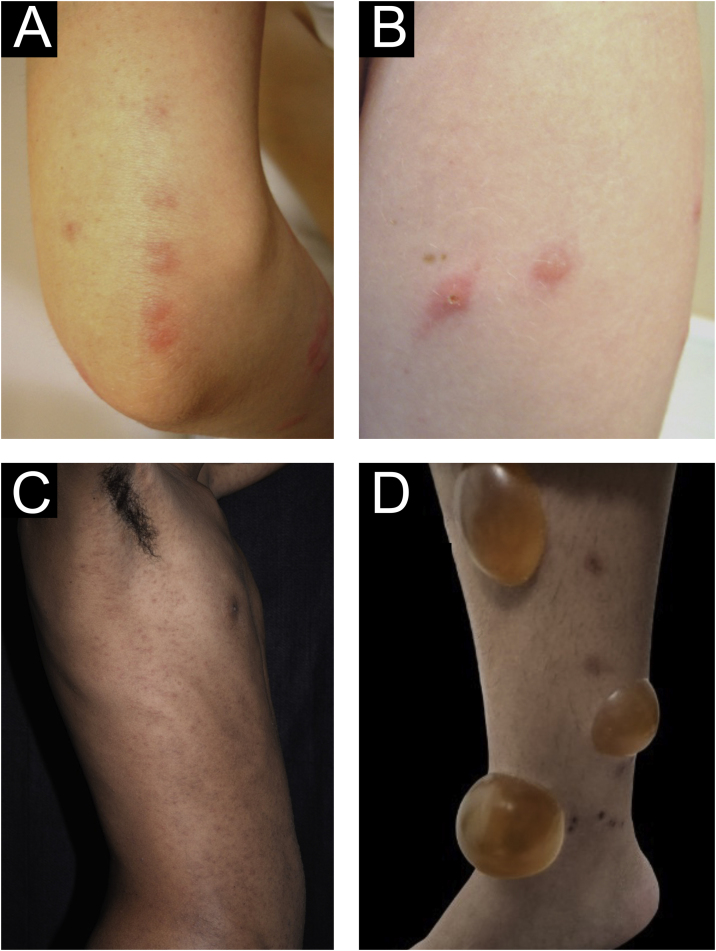
(A) Acute prurigo on the forearm due to bedbug bites in a typical linear pattern referred as the “breakfast, lunch and dinner” pattern; (B) Acute prurigo due to insect bites (prurigo strophulus); (C) Extensive acute prurigo due to scabies. (D) Bulla after acute prurigo due to insect bites.

Acute prurigo comprises lesions that last for a short time (≤ 6 weeks) with possible exacerbations. It comprises several clinical manifestations such as intensely pruritic erythematous papules, often showing a central vesicle, in areas covered by clothing (chest, abdomen), or mainly not covered, such as forearms, arms, legs, hands, and feet (Fig. 1), depending on the inducing agent. Atypical lesions may be associated, such as vesicles, bullae, crusts, and urticarial lesions, in which staphylococcal superinfection is common. Acute prurigo generally occurs due to arthropod bites and is commonly referred to in pediatric literature as prurigo strophulus, which favors children aged two to seven years, in tropical areas, with an atopic immunological background and low socioeconomic level.^2^, ^6^

The reactivation of old lesions after new insect bites in other topographies is described, as well as the formation of bullae and vesicles at the sites of new bites (Fig. 1). More exuberant lesions appear in predisposed individuals, especially atopic ones. The content of the lesions is usually citrine, reflecting the plasma present in the lesions.^7^

Acute prurigo usually does not require extensive additional examinations. Its treatment must be guided according to the causative agent. Treatment is symptomatic: non-sedating antihistamines (anti-H1) during the day and sedating ones at night, daily antiseptic care, nail cutting to avoid bacterial superinfection, and topical corticosteroids.^2^

However, exposure to intermittent arthropod bites can lead to a form of acute pruritus called papular urticaria (PU), which is mainly induced by flea bites.^8^

PU is a chronic allergic condition in which clinical improvement can occur around seven years of age, representing a natural model of acquired immunological tolerance. In patients with PU, immunological studies have demonstrated more skin-specific CLA4+ T-cells for flea antigens secreting IFN-γ, IL-4, IL-10, and IL-17 when compared to healthy controls.^8^, ^9^ Interestingly, after more than five years of disease duration, CLA4+ T-cells in the skin lose their ability to produce IL-4, while largely maintaining their capacity to secrete IL-10 and IL-17.^8^, ^9^ In line with this hypothesis, the frequency of T cells positive for IFN-γ is reduced in both resident and non-resident lymphocytes in the skin of these patients.^8^, ^9^

Celiksoy et al.^10^ studied 130 patients with PU (median age: 60 months). The prevalence of recurrent wheezing and atopic dermatitis was higher in the group under five years of age with PU than in the age-matched control group.^10^ The prevalence of asthma, allergic rhinitis, and atopic dermatitis was higher in the group over five years of age with PU than in the control group of the same age.^10^ These patients should be evaluated not only in terms of PU but also regarding comorbid atopic diseases.^8^, ^10^

### Chronic prurigo

Chronic prurigo (CPG) lasts for more than six weeks and can be classified into three subtypes, whether associated/caused by other dermatoses or not: (a) CPG with associated skin lesions, (b) CPG without associated skin lesions or (c) CPG of undetermined origin. A Japanese consensus classified the types of prurigo according to their related cause, as shown in Table 1.^1^

**Table 1:** Prurigo classification according to the underlying cause. Frequently, most of these forms develop into chronic prurigo.^16^

Insect bite reaction	
Symptomatic prurigo (associated with underlying systemic or cutaneous conditions)	Prurigo in atopic dermatitis
	Prurigo in diabetes mellitus
	Prurigo in renal failure
	Prurigo in liver failure
	Prurigo in internal malignancy
	Prurigo in hematological malignancy
	Prurigo associated with eczematous diseases
	Prurigo in HIV infection/ Pruritic-papular eruption of HIV (prevalence of 24%‒36%)
Prurigo in pregnancy/*Prurigo gestationis* (Special form: Polymorphic eruption of pregnancy)	
Prurigo in allergy to drugs	
Prurigo in allergy to metals	
Prurigo in psychiatric diseases	
Idiopathic prurigo (undetermined cause)	

CPG not associated with other skin lesions may be related to internal or systemic diseases.^2^, ^11^, ^12^ Table 2 shows dermatological diseases associated with CPG with or without skin lesions caused by other dermatoses.

**Table 2:** Dermatological diseases associated with chronic pruritus and prurigo, and chronic pruritus/prurigo without skin lesions from other dermatoses.^2^, ^11^

Dermatological diseases associated with chronic prurigo		Chronic prurigo without skin lesions from other dermatoses	
Inflammatory or parasitic skin diseases	Atopic dermatitis, lichen planus, scabies, lichen simplex chronicus	Infectious/parasitic conditions	Gastrointestinal parasitoses, chronic hepatitis, HIV infection
Drug-related prurigo	Contact eczema, Adverse drug reactions	Metabolic diseases or endocrinopathies	Acquired perforating dermatoses, chronic renal failure, cholestatic liver diseases, hyper/hypothyroidism, nodular amyloidosis
Autoimmune diseases	Autoimmune bullous dermatoses (dermatitis herpetiformis, bullous pemphigoid, linear IgA dermatosis, pemphigus vegetans)	Drugs	Angiotensin converting enzyme (ACE) inhibitors, angiotensin receptor antagonists, morphine, amiodarone, hydrochlorothiazide, allopurinol, etc.
Genodermatoses	Transient acantholytic dermatosis (Grover's disease), Darier's disease	Psychiatric	Psychotic or neurotic conditions
Neoplastic diseases	Cutaneous T- or B-cell lymphoma, leukaemia cutis	Neuropathic causes	Brachioradial pruritus
			Postherpetic neuralgia
			Polyneuropathy or mononeuritis
Psychiatric diseases	Pruritus associated with anxiety, depression, or other psychiatric conditions	Venous stasis	*Prurigo nodularis* due to the pruritus and scratching of varicose veins

**Figure 2:**
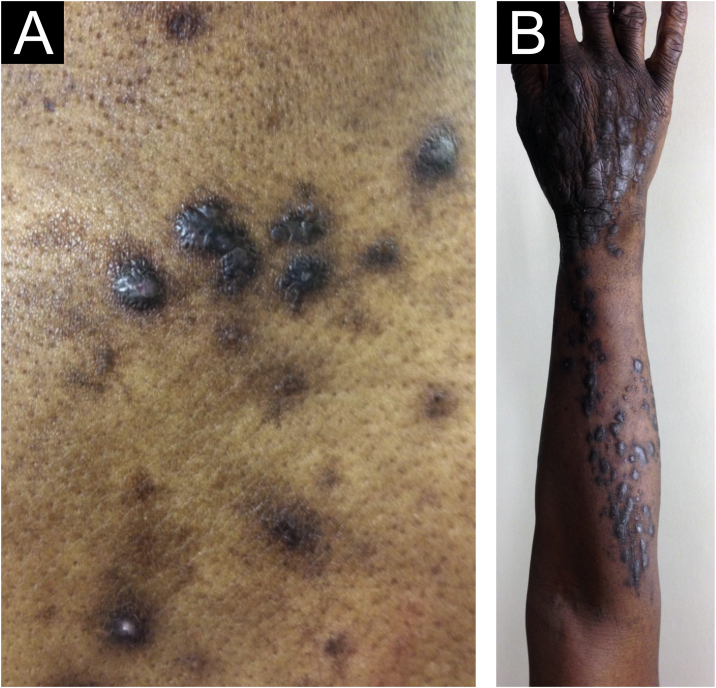
Chronic prurigo (CPG) and the elementary morphology of the lesions. (A) Papule (<1 cm in diameter) and nodules (>1 cm in diameter), (B) Coalescence of papules and nodules (plaque) and lesions in a linear arrangement.

Morphologically, CPG can be called chronic nodular prurigo (in which nodules predominate), chronic papular prurigo (where papules predominate), chronic prurigo in plaques (plaques predominate), chronic umbilicated prurigo (ulcerated pruritic lesions predominate) or linear prurigo (Fig. 2). However, as distinct morphologies can be present in the same patient simultaneously, and their characteristics can develop and change over time, all of these phenotypes will be referred as CPG in this text.^4^, ^13^, ^14^

Epidemiological data on CPG are scarce in the literature. Some authors estimate 72 cases per 100,000 individuals, a figure that is probably underestimated due to underreporting.^4^, ^14^ Docampo-Simón et al.^13^ reported that, in their clinical practice, CPG is more frequent in patients aged between 50 and 60 years and among African Americans.^14^

Zeidler et al. studied 1,128 CPG patients in Germany between 2004 and 2018, of which 61.4% were female, and observed that prevalence increased with age, and the median duration of CPG was 2.9 years before diagnosis.^15^

European and American pruritus experts developed the IFSI (International Forum for the Study of Itch) guideline on CPG, including prurigo nodularis as a synonym, and diagnostic criteria for CPG were established (Table 3).^16^ The Japanese definitions are slightly different.^1^ According to the IFSI etiological classification, CPG can also be of dermatological, systemic, neurological, psychiatric/psychosomatic, multifactorial, or unknown origin, showing the importance of the etiological investigation before treatment.^16^

**Table 3:** IFSI (International Forum for the Study of Itch) guideline on the diagnostic criteria for chronic prurigo. The three major criteria are necessary to establish the diagnosis of chronic prurigo. Minor criteria are often present but not mandatory.^16^

Major criteria		
(i) Chronic pruritus (≥ 6 weeks);		
(ii) History and/or signs of repeated pruritus;		
(iii) Presence of multiple localized or generalized pruritic lesions^a^		
**Associated criteria:**	(i) Clinical signs	Pruritic lesions: symmetrically distributed, rarely affecting the face and palms
		Signs of scratching: excoriation, scars, lichenification
	(ii) Diversity of clinical manifestations	Papular type
		Nodular type
		Plaque type
		Umbilicated type
		Linear type
	(iii) Symptoms	Usually pruritic lesions develop after the onset of pruritus
		Quality: pruritus, burning, pain or stinging
		Signs of chronicity: great intensity of pruritus, allokinesis, hyperkinesia, continuous increase in the number of lesions
		Impaired quality of life, loss of sleep, days of absenteeism from work and/or obsessive-compulsive behavior
	(iv) Emotions	Depression
		Anxiety
		Anger
		Disgust
		Shame
		Hopelessness

a Definition of pruritic lesions: excoriated papules and/or nodules and/or desquamative and crusted plaques, often with a whitish or pink center and hyperpigmented border.

Chronic pruritus induces a pruritus-scratching cycle, as well as neuronal sensitization phenomena that contribute to the development and perpetuation of CPG.^16^ These mechanisms are independent of the origin of the pruritus, as CPG development is observed in different underlying etiologies of pruritus, such as in atopic dermatitis (AD), nephrogenic pruritus, or neurological compression syndromes.^16^ In the elderly, many independent comorbidities may be present, but without representing a triggering cause for CPG.^16^ Therefore, the underlying CPG disease is not easy to establish and some terms such as “prurigoid atopic dermatitis” should be avoided in favor of stating that there are two distinct entities.^16^

## Pathogenesis of chronic prurigo

In atopic patients, immunological and genetic studies have outlined allergic inflammatory pathways common to several conditions, such as atopic dermatitis, asthma, and food allergy, among others, of which the central pathogenesis is the interleukin 4 receptor (IL-4R) pathway.^17^

The IL-4/IL-13/IL-4R axis promotes the differentiation of type 2 helper T cells (TH2), which mediate the pro-allergic adaptive immune response.^17^ Once IL-4 or IL-13 binds to the receptors, it triggers the transphosphorylation and activation of the Janus protein kinases (JAKs) family associated with the receptor subunit, including JAK1, JAK3, and JAK2.^17^ However, several other conditions are related to CPG, and their pathogenesis is based on pruritus sensitization and interactions between pruriceptors and skin cells.^18^

Pruritus involves structures and cells originating in neuroectodermal tissues. There are two main players involved in the pathogenesis of CPG: the central nervous system and skin immune cells.^18^

Brain functional changes occur, associated with sensitization to pruritus that show increased activation of some areas associated with structural changes, such as a decrease in gray matter in some brain regions.^16^

In the spinal cord, abnormal regulation of pruritus inhibitory pathways may explain central sensitization.^18^ Peripheral sensitization has been attributed to increased excitability of the sensory nerves due to hyperinnervation (in the dermis) and/or loss of innervation (intraepidermal small-diameter nerve fibers (IENFs), as well as increased expression, sensitivity, and/or response capacity of pruriceptors, which contribute to the hypersensitivity of sensory neurons to pruritogens.^10^

In the skin, keratinocytes interact with sensory nerve endings, especially IEFNs pruriceptors, through synaptic contacts.^18^ The perception of pruritus and the appearance of prurigo lesions are related to these interactions and with other cells in their environment, such as mast cells, eosinophils, dendritic cells, or T lymphocytes.^18^

Three types of pruriceptive nerve endings have been identified in mice: (a) MRGPRD (Mas-related G protein-coupled receptor) neurons; (b) Neurons expressing MRGPRA3, MMRGPRC11, histamine receptors, and the interleukin (IL)-33 receptor; and (c) Nerve endings that express serotonin receptors and the IL-31 receptor. Many MRGPR genes are expressed exclusively in subsets of small-diameter dorsal root ganglion (DRG) neurons, and in humans, proteins derived from these genes are referred to as MRGPRX1 to MRGPRX4.^19^ In humans, the MRGPR-X2 gene is highly expressed in CPG lesions, and mast cells are the main ones expressing MRGPRX2 mRNA in the majority of patients (70%).^20^ IL-4 and IL-13 receptors are detected in all three categories.^18^ In mice, scratching behaviors have been shown to vary depending on the activation of these different subtypes.^18^

In CPG, dermal expression of IL-31, IL-31RA Oncostatin M (OSM) and periostin are well correlated with pruritus intensity. Moreover, OSMRβ expression in the dermis is increased, although it is not correlated with the intensity of pruritus. IL-31 is associated with pruritus in several diseases, including AD psoriasis, cutaneous T-cell lymphoma, stasis dermatitis, bullous pemphigoid, scabies, and primary localized cutaneous amyloidosis. CPG expresses 50-fold more IL-31 mRNA in injured skin.^21^

IL-31 is mainly produced by activated Th2 cells. Epidermal keratinocytes, eccrine sweat glands, mast cells, basophils, eosinophils, and monocytes/macrophages are capable of releasing IL-31, and CD68+ macrophages, in addition to CD3+ T-cells are the main cell sources of IL-31 in CPG lesions. M2 macrophages express IL-31 and may be responsible for pruritus in several dermatoses, such as stasis dermatitis and scabies. Therefore, macrophages, together with T cells, maybe a potential therapeutic target for CPG-associated pruritus.^21^

Pruritus intensity is also closely related to the dermal expression of IL-31RA and OSMRβ (oncostatin M beta receptor).^21^ The largest population of IL-31RA+ dermal cells are macrophages and mast cells. OSMRβ+ dermal cells were mainly macrophages, whereas mast cells barely expressed OSMRβ. This indicates that macrophages can respond to IL-31, but mast cells cannot. The majority of cells infiltrating CPG lesions consist of macrophages, T cells, and mast cells. These cells are thought to cause skin inflammation and pruritus by secreting cytokines/chemokines and pruritogens (including IL-31) and, for macrophages, partially in response to IL-31 signaling involving IL-31RA and OSMRβ.^21^

OSM acts on dermal fibroblasts to release monocyte chemoattractant protein 1 (MCP-1), which can lead to the infiltration of monocytes/macrophages capable of releasing IL-31. These OSM functions may be involved in the pathogenesis of CPG and contribute to its association with pruritus. Epidermal keratinocytes have been reported to mediate IL-31-induced pruritus through the secretion of pruritogenic stimulators when stimulated by IL-31 through its receptor complex. The lower expression of epidermal OSMRβ may be related to the reduction of IEFNs, as insufficient OSMRβ could lead to the apoptosis of epidermal nerve fibers.^21^

The complex dermal milieu of immune cells/cytokines/receptor network including IL-31, OSM, IL-31RA, and OSMRβ may play an important role in the pathogenesis of pruritus in CPG.^21^, ^22^ However, in contrast to AD, M1/M2 macrophage activation, tumor necrosis factor production, dermal fibrosis, revascularization, and neural dysregulation are unique features of atopic CPG.^23^

A systemic and cutaneous Th22 immune polarization of CPG leads other participants to interact in its pathogenesis, as well as prominent dermal neuronal hyperplasia, where the neurite expresses CGRP and substance P.^21^ IL-4 induces the proliferation and migration of fibroblasts and stimulates the production of extracellular matrix proteins. Hyperactive IL-4 and IL-13 signaling have been implicated in the pathogenesis of fibrotic skin diseases. IL-4 and IL-13 positively regulate the promoter activity and transcription of pro-fibrotic genes, such as type I collagen and TGF-β. Increased dermal periostin and decreased epidermal periostin were also found in CPG patients compared to healthy controls.^24^

Moreover, there is some relevance regarding the involvement of the microbiome in patients with CPG, especially the role of *S. aureus*.^25^, ^26^ Additionally, thymic stromal lymphopoietin (TSLP) induces skin hyperplasia by activating immune cells, promoting proliferation of keratinocytes (directly), interrupting keratinocyte differentiation and increasing the expression of pro-inflammatory mediators, contributing to the hyperplastic phenomenon.^27^

IL-22 is involved in several chronic inflammatory conditions, such as coronary artery disease and type II diabetes mellitus (T2DM), both of which are highly associated comorbidities in patients with CPG.^23^ In the epidermis, high levels of IL-22 promote keratinocyte hyperplasia and acanthosis, in addition to acting synergistically with IL-17, which promotes keratinocyte hyperplasia and impaired epidermal differentiation in CPG.^23^

An increase in IL-31 expression is observed in Europeans when compared to African Americans; however, upregulation of Th22-related cytokines in CPG may be independent of ethnicity.^23^ In contrast, an increased expression of Th2 cytokines, including IL-4, IL-5, and IL-13, as well as greater Th2 activation was observed in Europeans but not in African-Americans.^23^ This cytokine profile may be the key to successful responses to treatment with specific drugs, such as dupilumab, which may be more effective in certain endotypes of CPG patients with associated atopy, involving IL-4Rα, and/or nemolizumab in patients of European descent. The IL-31 receptor consists of the IL-31 receptor alpha (IL-31RA) and oncostatin M receptor beta (OSMRβ), which depend on JAK1/2,^24^ and indicate a window of opportunity for JAK1 selective drugs such as abrocitinib and upadacitinib in the treatment of CPG.^28^

## Diagnosis of chronic prurigo and its evaluation

The diagnosis of CPG is made clinically based on clinical history, and clinical, histopathological, laboratory, and radiological examination, which help to confirm the diagnosis and determine disease severity, the underlying disease, and an individualized treatment plan.^16^

Anamnesis of patients with CPG should involve several objective questions.^16^ The general physical and dermatological examination suggested for CPG, complementary laboratory and imaging tests recommended by the IFSI guideline in CPG,^16^ with adaptations, based on clinical practice, are listed in Table 4.

**Table 4:** Proposal for dermatological and laboratory examinations in patients with chronic prurigo.^16^

I. PHYSICAL EXAMINATION:
**1. Dermatological examination:**
- Clinical distribution of pruritic lesions (e.g., localized/generalized; symmetrical/asymmetrical)
- Dermatological morphology of pruritic lesions;
- Number of affected body regions; number of pruritic lesions/with excoriated lesions on top
- *Butterfly sign* (area spared from lesions or excoriations on the back, where the patient's hands cannot reach)
**2. Objective methods:**
- Measurement of pruritic lesions (with a ruler or another equipment);
- Monitoring a target lesion when possible (clinically or through photographs);
- Photographic documentation;
- Scores: IGA^a^, for the staging of chronic prurigo
**3. Overall physical examination:**
- Abdominal palpation, assessment of lymphadenopathy, assessment of muscle strength, auscultation (lungs, heart, abdomen)
- Simple neurological examination;
- Dermatological examination: presence of concomitant skin diseases, xerosis, inspection of mucous membranes;
- Dermoscopy of pruritic lesions (searching for ectoparasites such as *Sarcoptes scabiei*);
- Dermographism assessment; Nikolsky sign testing; Darier sign testing.
**II. SKIN BIOPSY/FLUIDS and BLOOD EXAMS:**
1. Skin biopsies
2. Fluids/microbiological culture when there are signs of infection
3. Direct microscopic evaluation: screening for *Sarcoptes scabiei*
- Direct and indirect immunofluorescence (when autoimmune conditions such as bullous pemphigoid, dermatitis herpetiformis, linear IgA dermatosis, paraneoplastic pemphigus, etc. are suspected).
- Histopathology (hematoxylin-eosin staining or histochemistry for amyloid, mucin and other cutaneous deposits)
**III. COMPLEMENTARY DIAGNOSTIC EXAMS**
**1. Avoid invasive diagnostic procedures, if unnecessary, for:**
- CPG with pruritus of mild intensity or small extent;
- CPG of non-dermatological origin;
- Elderly, pediatric and frail patients.
**2. Groups of patients in whom imaging and laboratory studies are recommended:**
- With a family history
- With a medical history (e.g. presence of bloody sputum, change in cough profile)
- Rule out underlying diseases in all patients with CPG
- In all patients with CPG duration of at least one year
- In patients with CPG who report night sweats, or weight loss
- In patients with CPG of undetermined origin
- In CPG patients with disproportionate pruritus
- When patients demand laboratory tests
**3. Regularly performed laboratory tests:**
- Blood count; electrolytes, ESR, CRP, fasting blood glucose, glycated hemoglobin (HbA1c), iron, ferritin, LDH, TSH
- Total serum IgE, specific IgE (if respiratory or ocular allergy is suspected)
- Renal retention parameters (e.g., potassium, creatinine, urea, glomerular filtration rate)
- Liver enzymes (e.g., gamma-GT, AST, ALT, alkaline phosphatase, bilirubin)
**4. Laboratory tests - based on pathological findings or medical history :**
- Serum and urinary protein electrophoresis, immunofixation (increased sensitivity if monoclonal gammopathy is suspected)
- Flow cytometry immunophenotyping for hematological malignancies in peripheral blood^b^
- Serum tryptase (if systemic mastocytosis is suspected)
- Diagnosis of thyroidopathy in special situations (e.g., parathyroid hormone, phosphate, Ca2+, fT3, fT4).
- Vitamin B12, folic acid (if anemia is present).
**5. Imaging exams, if the history and physical examination are suggestive of neoplasia or systemic diseases:**
- Computed tomography (CT) of the skull, chest and abdomen; digestive endoscopy, colonoscopy

a IGA, Investigator Global Assessment.

b Immunophenotyping by flow cytometry is indicated in the following clinical situations: cytopenia, especially bicytopenia and pancytopenia; elevated white blood cell count, including lymphocytosis, monocytosis and eosinophilia; presence of atypical cells or blasts in peripheral blood, bone marrow or body fluids; plasmacytosis or monoclonal gammopathy; and organomegaly and tissue masses.^21^

## Chronic prurigo variants

In Japanese patients, in addition to CPG/prurigo nodularis (Hyde), prurigo chronica multiformis (PCM) is reported, first described by Lutz in 1957, as a chronic, intensely pruriginous skin disorder that mainly affects elderly individuals.^29^

**Figure 3:**
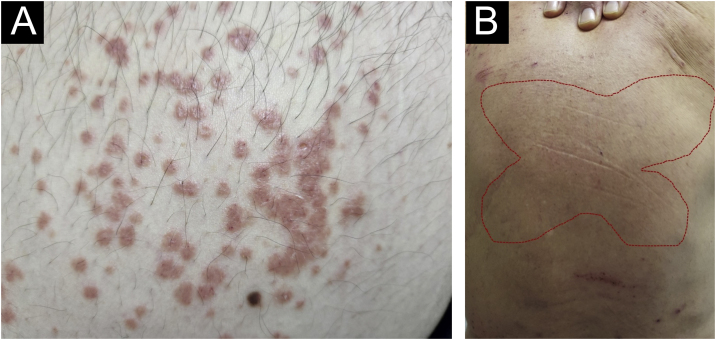
Prurigo chronica multiformis: (A) Multiple primary pruritic papular lesions usually expand or coalesce to form an infiltrated erythematous plaque lesion; (B) the “butterfly” sign, indicating an area of dorsal skin spared by excoriation due to inaccessibility.

There has always been confusion in the synonymy and classification of pruritic lesions, and Greither included PCM as a variant of subacute prurigo, with a trace of neurodermatitis.^20^, ^30^ The characteristic morphology, predilection site, age, and histopathological findings of PCM indicate that it is a distinct dermatosis and not a variant of subacute or chronic prurigo simplex (Fig. 3).^29^ The “butterfly sign” is an example of a “sparing phenomenon” that represents an area of normal or relatively hypopigmented thoracolumbar skin that is spared from scratching due to its inaccessibility (Fig. 3), in patients with generalized pruritus due to many disorders such as hepatobiliary disease, atopic dermatitis, and CPG.^31^

Fujii et al.^29^ collected more than 30 cases of PCM from 1997 to 2000, according to the following criteria: (i) The primary lesion starts as an intensely pruriginous, solitary papular or pruritic lesion that develops on the lateral side of the chest, lumbar region, buttocks and extensor surfaces of the thighs; (ii) The upper back, shoulders, extensor surfaces of the upper extremities, lower abdomen, anterior chest and calves may also be affected; (iii) Primary pruriginous papular lesions often expand or coalesce to form an infiltrated erythematous plaque; (iv) The coalescent nature of the primary eruption differentiates PCM from usual chronic prurigo or subacute prurigo; (v) Intense pruritus often causes focal areas of acute eczema on and around plaque lesions, but sero-papular and/or desquamative components never dominate the cutaneous manifestations; (vi) An infiltrated plaque-like lesion extends beyond the confines of the skin area, which is clinically different from the lichenification of chronic eczema or neurodermatitis; (vii) On histopathological examination, both primary pruriginous papular lesions and plaque lesions show moderate degrees of perivascular infiltration of mononuclear cells containing varying populations of eosinophils in the reticular and upper papillary dermis, (viii) Unlike the primary lesions of subacute prurigo simplex, the epidermis does not always show spongiosis with exocytosis, suggesting that epidermal spongiosis should not be the main pathological alteration of the disease.^29^

Thus, this unique variant of chronic prurigo is characterized by urticarial or solid pruriginous papules, which sometimes coalesce to form lichenified plaques; the lesions are spread over the trunk and extremities with each eruption lasting several weeks (Fig. 3).^32^ PCM occurs more frequently in men and older patients, unlike CPG, which occurs more in women and middle-aged patients, in Japan.^32^

Inui et al.^32^ studied 168 cases of chronic prurigo in Japan (103 with CPG and 65 with PCM) and demonstrated that in both CPG and PCM, serum IgE levels tended to be high (> 256 IU/mL), suggesting the involvement of immune Th2 responses in chronic prurigo. These authors found that serum levels of TARC/CCL17 and the number of blood eosinophils were higher in PCM than in CPG. Moreover, the distribution of eosinophils was also distinct in CPG and PCM, but their density was comparable in both groups. These findings suggest that increased levels of eosinophils in peripheral blood and the recruitment of these cells to collagenous areas of the dermis may be important for the pathogenesis of PCM.^32^

PCM is often resistant to treatment with a combination of topical steroids and antihistamines, particularly when the skin lesion is actively expanding.^32^

All forms of CPG have the hallmarks of intense pruritus and lesions secondary to the act of scratching.^10^ Although CPG is a specific clinical entity, it can also be secondary to several underlying conditions.^33^ CPG has a significant impact on quality of life,^11^ greater than other skin conditions where pruritus is a prominent feature, such as psoriasis or atopic dermatitis.^14^ Anxiety occurs in 37% of patients, depression in 29%, and suicidal ideation in 19%.^13^, ^14^

Experts belonging to the European Prurigo Project published a consensus in 2018 to propose definitions, classifications, and terminology.^4^, ^13^ The article states that CPG is “a distinct disease defined by the presence of chronic pruritus and multiple localized or generalized pruriginous lesions”. Sensitization to pruritus and the development of a pruritus-scratch cycle may be of dermatological, systemic, neurological and psychiatric/psychosomatic origin, multifactorial or undetermined”.^4^, ^13^

## Histopathology of chronic prurigo

**Figure 4:**
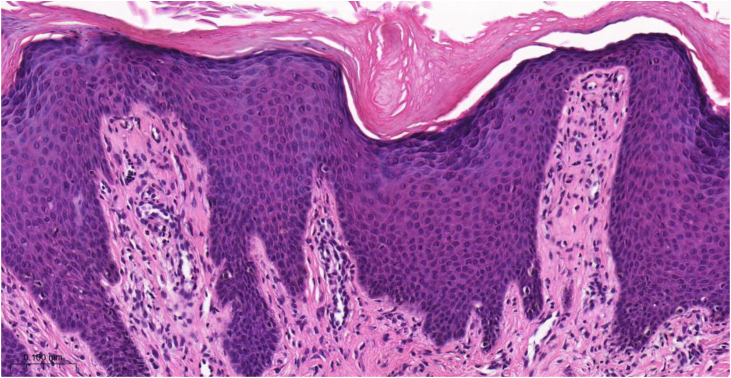
Histopathology of chronic prurigo showing hyperkeratosis with sparse parakeratosis, irregular acanthosis, discrete spongiosis, vascular prominence in the dermal papillae, fibroplasia of the superficial dermis and lymphocytic inflammatory infiltrate, mostly perivascular (Hematoxylin & eosin, ×400).

The histopathological examination of CPG nodularis skin lesions under light microscopy may vary depending on the stage and severity of the lesion and the individual patient response to scratching and chronic irritation. Hyperkeratosis, excoriation, acanthosis (sometimes psoriasiform), mild spongiosis, dermal fibrosis, and mild perivascular infiltrate (scarce lymphocytes and eosinophils) underlie the disease (Fig. 4).

An increased density of dermal nerve fibers and changes in many skin cell types, including mast cells, Merkel cells, epidermal keratinocytes, dendritic cells, endothelial cells, and collagen fibers are also reported.^34^ These cells cause inflammation and pruritus through the release of tryptase, interleukin-31 (IL-31), prostaglandins, cationic eosinophil protein, histamine, and other mediators such as neuropeptides, including substance P, calcitonin gene-related peptide (CGRP), and growth factor.^34^, ^35^

## Treatment of chronic prurigo

Treatment of prurigo can be challenging due to the recurrent history of the lesions, caused by underlying diseases (e.g., AD, uremia), or due to the limited use of systemic medications in patients susceptible to adverse effects (e.g., elderly individuals). Table 5 lists a series of approaches, either pharmacological or not, reported in the literature for the treatment of pruritic lesions; however, only the correct diagnostic classification, and identification of underlying conditions, when carefully investigated (Table 4) can lead to greater therapeutic success.

**Table 5:** Treatments indicated for prurigo.

**Topical treatments**	Emollients
	Topical corticoids^a^
	Calcineurin inhibitors (tacrolimus/pimecrolimus)
	Intralesional corticoids^a^
	Topical capsaicin^a^
**Systemic treatments**	UV Phototherapy (narrow band UVB)
	H1 antihistamines
	Gabapentin, pregabalin a
	Antidepressants (e.g., mirtazapine)^a^
	Cannabinoids
	Immunosuppressants (methotrexate, cyclosporine A)^a^
	Thalidomide^a^
	Neurokinin 1 antagonist (NK1 ‒ aprepitant)^a^
	μ-opioid receptor antagonist (naloxone, naltrexone)^a^
	JAK inhibitors (abrocitinib, tofacitinib, upadacitinib)^a^
	Anti IL-4/13: Dupilumab^a^
	Anti-IL-31: Nemolizumab^a^
	Anti-oncostatin, anti-receptor (OSMRbeta - IL-31): Vixarelimab^a^
	KIT anti-receptors (CDX-0159)^a^

a Indicated in chronic prurigo.

Overall, the prescription of emollients, topical antipruritic agents, topical corticosteroids, calcineurin inhibitors, and non-sedating antihistamines are not very effective in CPG, while sedating antihistamines can lead to adverse effects, especially in patients who require professional attention (e.g., drivers), or elderly individuals who accumulate anticholinergic effects. Moreover, the pathogenesis of CPG is complex, and histamine is not among the main mediators involved.

Usually, the treatment of CPG depends on systemic medications such as corticosteroids, cyclosporine, mycophenolate mofetil, azathioprine, thalidomide and neuromodulators (e.g., gabapentin or pregabalin); in addition to phototherapy (especially narrow-band UVB). However the demand for prolonged use of these options maximizes adverse effects.

Dupilumab is a human IgG4 monoclonal antibody (mAb) that binds to IL-4Ra and inhibits IL-4R signaling induced by IL-4 and IL-13 and downregulates TH2 inflammation in atopic dermatitis and other inflammatory and atopic diseases.^17^ It has an antipruritic effect in patients with CPG. Two recent phase 3 studies also demonstrated that treatment with dupilumab reduced pruritus and skin lesions in patients with CPG, with the indication for “prurigo nodularis” being incorporated into the directions on the medication in Brazil.^36^

**Figure 5:**
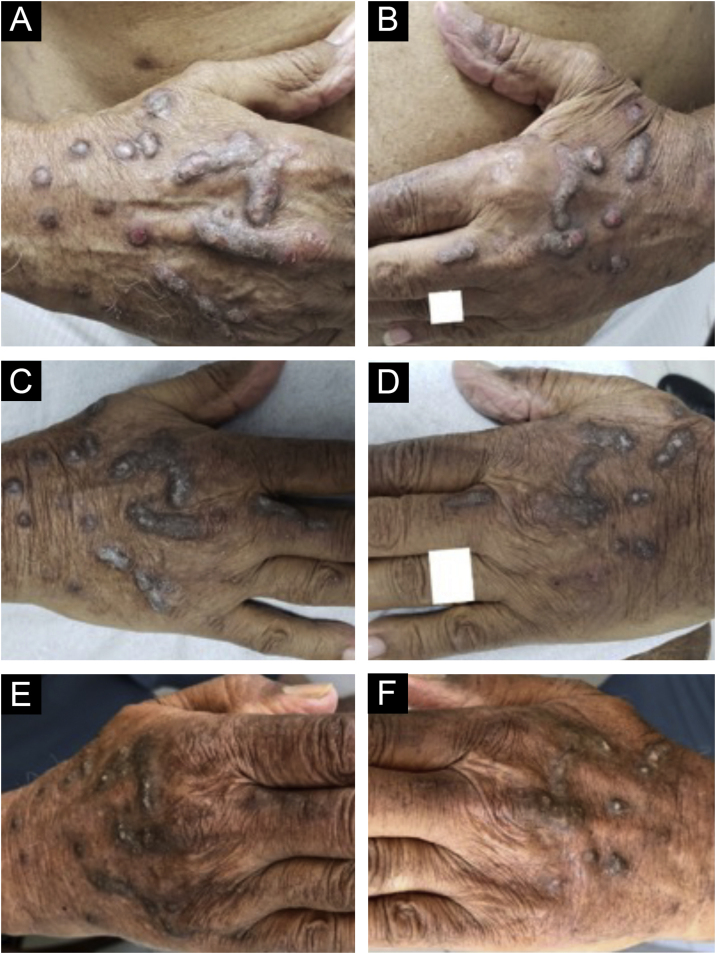
Evolution of chronic prurigo nodular lesions in an 87-year-old man before (2019) and after treatment with dupilumab. Sequential evolution of isolated or confluent infiltrated and excoriated hyperkeratotic nodules on the patient's hands. (A) Right aspect and (B) left aspect at initial consultation (note erosions and excoriations over nodules). (C) and (D), fewer excoriated lesions at the end of the first month of treatment with dupilumab; (E) and (F), after four months of dupilumab monotherapy, the infiltrated and hyperkeratotic nodules showed significant regression and no erosions or excoriations were observed. After three years of monitoring the treatment with dupilumab (2019‒2022), the patient turned 91 years old, using 300 mg once a month, and denied pruritus with the Visual Analogue Scale (VAS) for pruritus constantly 0 for the last two years.

Dupilumab therapy reduced the signature of more than 800 genes affected in atopic dermatitis.^37^ Dupilumab decreases the mRNA expression of genes related to epidermal hyperplasia (K16 and MKI67), T cells, and dendritic cells (CD1b and CD1c), and potent inhibition of chemokines associated with the TH2 pathway (CCL17, CCL18, CCL22, and CCL26) was observed without significant modulation of genes associated with the TH1 pathway (IFNG).^37^ These actions may explain the good response of dupilumab in the treatment of CPG (Fig. 5).

Chronic AD skin lesions present increased infiltration by T cells, dendritic cells (DC), and eosinophils^17^; similar to what is found in CPG lesions. Although AD has been classified as a TH2-dominated disease, other T cell subsets (TH22, TH17, and TH1 cells) may also contribute to the pathogenesis. As mentioned above, some authors found an upregulation of IL-31 mRNA in CPG.^35^ The binding of IL-31 to its receptors activates powerful signaling pathways (i.e., activation of Janus kinases JAK1 and JAK2 and initiation of the JAK-STAT pathway). Thus, IL-31 was considered a new player in type 2 inflammation.

This theory was corroborated by the discovery that Th2 cells are one of the main producers of IL-31, with a positive correlation between IL-31 and AD severity. Moreover, elevated levels of IL-31 contribute to specific symptoms such as pruritus, skin lesions, and localized irritation/inflammation in AD^17^ and endothelin axes in lesional and perilesional skin in CPG. Zhong et al. observed higher expression of IL-31 mRNA in the lesions of five atopic patients than in six non-atopic ones. These results suggest a much more important role for IL-31 in patients with atopic *versus* non-atopic CPG.^38^

CPG is an orphan disease, with high rates associated with anxiety, depression, and a high impact on quality of life. Chronic pruritus also has a negative impact on sleep and quality of life.^39^ Zhai et al., in a retrospective study involving 20 patients with chronic recalcitrant pruritus (nine with CPG; five with uremic pruritus; four with chronic idiopathic pruritus; one with lichen planus and one with eosinophilic dermatosis related to hematological malignancy) demonstrated the off-label use of dupilumab with a successful outcome in reducing pruritus in all patients, leading to complete resolution in 12/20 patients, and an overall mean numerical rating scale for pruritus intensity (NRSi) of 7.55.^40^ Dupilumab was well tolerated without major adverse effects in the elderly.^41^

Of ninety adult patients with AD treated with dupilumab^41^ in two Italian university hospitals, 10.0% had generalized CPG and were affected by one or more atopic comorbidities (allergic rhinitis, asthma, and allergic conjunctivitis). The serum IgE level was above normal in all patients. The nine CPG patients were treated with dupilumab using the standard dose regimen. After 16 weeks of treatment, all patients showed marked clinical improvement, and none of the patients abandoned treatment.^41^

The therapeutic approach for CPG is also based on case series and clinical experience: corticosteroids, phototherapy, topical immunomodulators (topical calcineurin inhibitors) and immunosuppressants (cyclosporine, azathioprine, mycophenolate mofetil and methotrexate),^42^ neuromodulators and thalidomide, however with significant potential undesirable side effects, especially in elderly patients. Systemic agents are often required, including intralesional corticosteroids, antipruritic agents such as oral antihistamines, neuromodulators such as gabapentin and pregabalin, and phototherapy.^43^, ^44^, ^45^

In more severe cases, immunosuppressive agents have been used with variable success and a number of undesirable side effects.^43^, ^44^ Another aspect of the use of systemic immunosuppressants in tropical countries is endemic infectious diseases, such as gastrointestinal parasites (strongyloidiasis prophylaxis is mandatory), and the risk of elderly patients developing severe endemic infectious diseases such as dengue fever, zika, and chikungunya^46^ or new epidemic viruses such as COVID-19.^47^

Overall, a rational approach to treating CPG is possible by taking five steps into consideration. The first step is to use topical corticosteroids, calcineurin inhibitors (tacrolimus and pimecrolimus), and ultraviolet phototherapy (narrowband 311 nm, excimer laser, and PUVA therapy). The second step involves the use of topical capsaicin at concentrations ranging from 0.025% to 0.3% four to six times a day, in addition to the oral use of gabapentin 300-900 mg/day up to three times a day or pregabalin 150-600 mg/day divided into two to three doses. The third step comprises using antidepressants, such as paroxetine 20‒50 mg/day or mirtazapine 15 mg/day. The fourth step, for cases in which topical control and antidepressants and/or gabapentinoids have not resulted in improvement, is the use of immunosuppressants such as cyclosporine and methotrexate. For more refractory cases, the fifth step would also include the use of µ-opioid receptor antagonists, such as naltrexone; and neurokinin 1 receptor antagonists.^48^ As atopic dermatitis is associated with half of CPG cases, there should be a convergence in the treatment of these two conditions, including the use of biologicals such as dupilumab and nemolizumab, and small molecules like JAK inhibitors; for example, upadacitinib, baricitinib, tofacitinib, and abrocitinib.^49^

Dupilumab is well tolerated by children, adults, and the elderly, with few serious adverse events, which include nasopharyngitis, headache, conjunctivitis and injection site reactions, alcoholic flush in one case, transient skin erythema and desquamation, local site reactions, herpes simplex infections, and alopecia.^50^ Special attention should be given to prescribing dupilumab in patients with cutaneous T-cell lymphoma (mycosis fungoides), due to the risk of unfavorable evolution of the underlying disease.^51^

New emerging drugs appear as promising options, such as nemolizumab, a humanized antibody against the IL-31-α receptor (IL-31Rα). IL-31 is a pro-inflammatory cytokine that is elevated in several chronic pruriginous dermatoses, and the blocking of its receptor has recently shown a reduction in the pruritus score in patients with CPG, improved quality of life and sleep, in comparison to placebo. in a 12-week randomized double-blind study.^52^ The adverse events reported in this study were mild and uncommon, mainly diarrhea, abdominal pain, and musculoskeletal symptoms. Another study evaluated the transcriptome of patients with CPG after nemolizumab therapy, showing downstream inflammatory factors after 12 weeks of treatment, as well as changes in the transcriptome.^53^ Due to this evidence, the FDA recently considered nemolizumab a “breakthrough therapy” for patients with CPG. Further studies are required to ensure the efficacy and safety of the IL-31Rα inhibitor in patients with CPG and, at this time, there are four ongoing clinical trials.^54^, ^55^, ^56^, ^57^

Other biologicals and small molecules are currently being investigated, such as IL-4 and IL-13 inhibitors, anti-OSMβ receptors, KIT tyrosine kinase receptor inhibitors, and Janus kinase (JAK) inhibitors.^58^, ^59^, ^60^, ^61^ Tralokinumab, an IL-13 inhibitor, showed improvement in an open-label study in atopic patients with CPG.^61^

**Figure 6:**
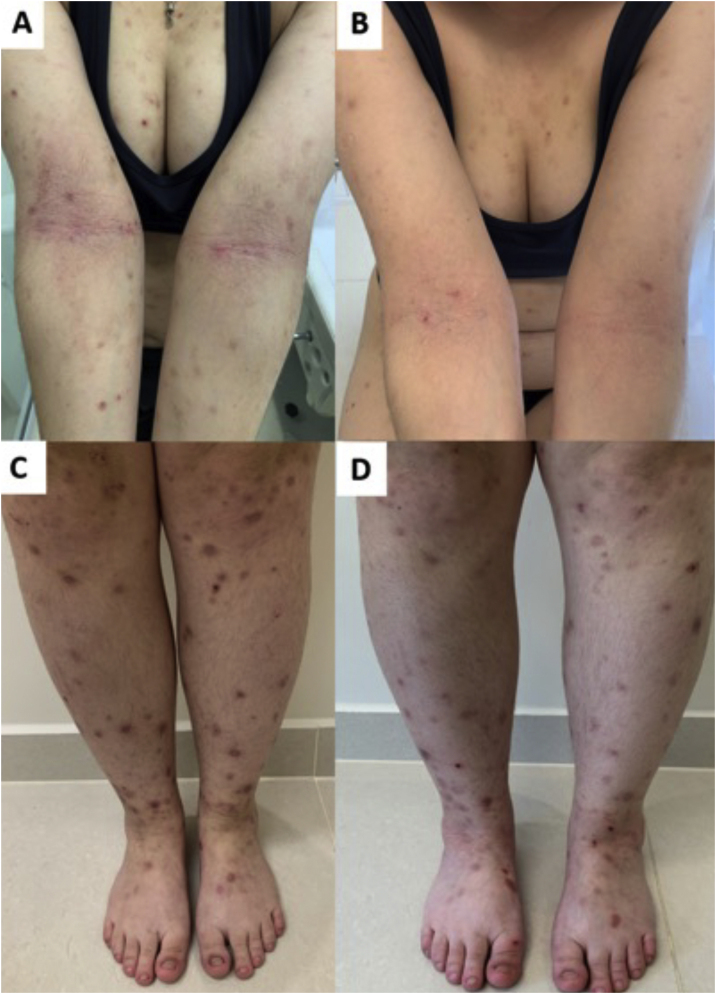
Response of prurigo nodularis to upadacitinib. (A) Woman with eczematous lesions of classic atopic dermatitis in the antecubital folds and prurigo nodularis nodules on the arms and lower limbs (C). (B and D), after 12 weeks of treatment with upadacitinib 30 mg/day, there was significant improvement in the eczematous lesions, pruritus and, consequently, prurigo.

JAK inhibitors have more recently emerged as another option for recalcitrant CPG by inhibiting the JAK-STAT pathway, blocking IL-4 and IL-31 transcription, increasing epidermal nerve fiber density, and reducing pruritus (Fig. 6). The JAK-STAT pathway positively regulates numerous cytokines and CD4 cells and has been implicated in the pathogenesis in other inflammatory skin conditions, such as atopic dermatitis (AD).^62^, ^63^ Case reports of the efficacy of tofacitinib, a non-selective inhibitor of JAK1 and JAK3, have been described.^64^ Topical 2% tofacitinib also demonstrated a substantial reduction in pruritus in two patients with CPG.^65^ Abrocitinib, a JAK1 inhibitor first studied in AD, is currently being studied for CPG in clinical trials.^66^ A recent study compared 36 and 13 patients (with or without AD) using dupilumab and oral JAK inhibitors (baricitinib and upadacitinib), respectively. Both groups achieved similar pruritus responses (WI-NRS), while IGA PN-S scores of 0 or 1 were achieved by 40.0% of dupilumab and 25.0% of JAK inhibitor patients.^67^

Better knowledge of the pathogenesis, associated with the availability of a range of new treatments, should change the history of the disease and the scenario for patients with CPG.

## Conclusions

Several diseases manifest clinically as pruritic lesions, and the correct clinical diagnosis must precede premature treatment. Furthermore, CPG represents a recalcitrant and distressing dermatosis, and at least 50% of these patients have atopic diathesis, the treatment of which can induce adverse effects, especially in the elderly. Quality of life is significantly compromised, and topical treatments are often unable to control symptoms and skin lesions. Systemic immunosuppressants, immunobiological and JAK inhibitors, despite the cost and potential adverse effects, may be necessary to control CPG and offer clinical and quality of life improvement for these patients.

## Financial support

None declared.

## Authors' contributions

Paulo Ricardo Criado: Design of the study, writing and approval of the final version of the manuscript.

Roberta Fachini Jardim Criado: Design of the study, writing and approval of the final version of the manuscript.

Mayra Ianhez: Design of the study, writing and approval of the final version of the manuscript.

Juliana Nakano: Design of the study, writing and approval of the final version of the manuscript.

Daniel Lorenzini: Design of the study, writing and approval of the final version of the manuscript.

Hélio Amante Miot: Design of the study, writing and approval of the final version of the manuscript.

## Conflicts of interest

Paulo Criado: Advisory board - Pfizer, Galderma, Takeda, Hypera, Novartis, Sanofi; Clinical research - Pfizer, Novartis, Sanofi, Amgen and Lilly; Lecturer: Pfizer, Abbvie, Sanofi-Genzyme, Hypera, Takeda, Novartis.

Roberta Fachini Jardim Created: Advisory board - Pfizer, Takeda, Hypera, Novartis, Sanofi; Clinical research - Pfizer, Novartis, Sanofi and Lilly; Lecturer: Pfizer, Abbvie, Sanofi-Genzyme, Hypera, Takeda, Novartis.

Mayra Ianhez: Advisory Board - Galderma, Sanofi, Pfizer, Novartis, Abbvie, Janssen, UCB-Biopharma, Boehringer-Ingelheim; Lecturer - Galderma, Sanofi, Pfizer, Theraskin, Novartis, Abbvie, Janssen, Leopharma, FQM.

Juliana Nakano: Advisory Board - Novartis, Abbvie, Janssen, Boehringer-Ingelheim; Lecturer - Sanofi, LeoPharma, Lilly, Novartis, Abbvie, Janssen, Boehringer-Ingelheim.

Daniel Lorenzini: Advisory Board - Abbvie, Sanofi, Galderma; Lecturer - Sanofi, Abbvie, Pfizer, Lilly, Leopharma.

Hélio Miot: Advisory Board – Johnson & Johnson, L’Oréal, Theraskin, Sanofi and Pfizer; Clinical research - Abbvie, Galderma and Merz.
